# Varietal evaluation of hybrid maize in the summer and winter seasons in terai region of Nepal

**DOI:** 10.1016/j.heliyon.2022.e11619

**Published:** 2022-11-16

**Authors:** Sandesh Thapa, Sara Rawal, Sandhya Adhikari

**Affiliations:** a Gokuleshwor Agriculture and Animal Science College, Baitadi, Nepal; b Institute of Agriculture and Animal Science, Paklihawa Campus, Paklihawa, Nepal

**Keywords:** Genotype x environment interaction, AMMI analysis, Corn breeding, Heritability, Winter, Summer

## Abstract

Comparing hybrid maize to open-pollinated varieties, the former is widely recognized for its higher producing capacity. However, the production potential of hybrids varies depending on the region and the season. In order to find the high yielding stable genotypes throughout both settings, this experiment was carried out in the summer of 2021 and the winter of 2021–2022, using a Randomized Complete Block Design with 11 genotypes and 3 replications. The tallest genotypes among those evaluated were CP 808 and G-25 in the winter and summer, respectively. Both P3553 in the winter and CP 808 in the summer reported earlier days to anthesis. Grain yield showed a substantial and positive links with cob characteristics and a negative correlation with reproductive traits, according to correlation analysis. When compared to the yield of both seasons, P3355 and Bisco gold 941 showed highest yield in both environments. Thus, genotypes P3355 and Bisco gold 941 are recommended for cultivation in study area over other available hybrids in the market.

## 1 Introduction

Maize is the major cereal of Nepal both in terms of production and use ranking second in production after rice and being widely used for human consumption and for agro-industries (feed industry for poultry, fish and cow) [1]. Its cultivation is diverse, ranging from Terai and Inner- Terai to the hilly regions of the country. Hills practice rainfed farming whereas irrigated conditions prevail in the Terai and Inner- Terai regions of Nepal. Mainly maize is sown in spring season [2] after harvesting of wheat in mid-hills and both in spring and summer in Terai region of Nepal [3, 4].

The use of Open Pollinated variety (OPV) is high in mid-hills but in Terai region, hybrid maize tends to be dominating with increase in yield per hectare. There are several maize varieties available in Nepal's seed market from different international corporations, some of which are registered and some of which are not. This leads to a variety of issues in Nepal, from barren cob to a high disease incidence and low plant stand [5]. Hybrid maize has potential of giving 20–30% more grain yield in comparison to other cultivated maize varieties. In rice-wheat based farming system with irrigation facility, hybrid maize is best option for catch crop in mid hill and Terai [5].

The study of genotypes in different seasons is desired as a genotype suitable for summer cultivation may not be suitable for winter and vice versa and some can perform optimally with stable production in both seasons [3, 4, 6]. The variation in yield and yield attributing traits of maize has also been associated with variation in agro-climatic conditions [7, 8] and cropping domain [2, 5].

Though there are 45 registered commercial maize hybrids in Nepal, the hybrids that performs better with stable grain yield in individual and across the season is not studied. Kandel and Shrestha [2] reported that Shresta and P3396 were best yielding genotype in two year when sown in winter season.

Due to fragmentation, the majority of farmers in developing nations have extremely limited access to land, and the hybrid maize seed that is available is rarely used entirely in a single cropping season. Therefore, small landholder farmers may find it advantageous to adopt high yielding maize cultivars in both seasons, improving the productivity of the crop, if we were able to identify high yielding and stable varieties/hybrids for both the summer and winter seasons. The demand of hybrid seeds of maize is almost dependent on India with very few national hybrids (Released by National Maize Research Program (NMRP), Chitwan) under cultivation, among which Rampur hybrid-10 is one of the most promising heat resilient maize hybrid [2, 5]. Due to open border with India, hybrid seeds enter Nepalese market and farmers field without any certification [9]and registration procedure which have caused huge difficulty in relation to production, incidence of disease and pest and crop failure at early stage [10]. Nowadays, NMRP is conducting multi location, multi environment and multi-year trials to identify the stable, high yielding and promising maize genotypes [11]. Followingly, it is also conducting performance trial of various multinational companies and their registration for Nepalese market [11]. Large number of genotypes were suggested for cultivation but they donot perform optimally in all environment [2, 5]. Thus, genotypes are recommended for diverse ecological condition rather than specific ecological condition. Thus, this study was conducted as per farmers demand in Sunsari district with an objectives identify the high yielding maize for cultivation in both the seasons to study genetic variability, correlation and AMMI analysis for yield and yield related traits that would aid in crop improvement programs.

## 2 Materials and methods

### 2.1 Experimental location details

**Figure 1:**
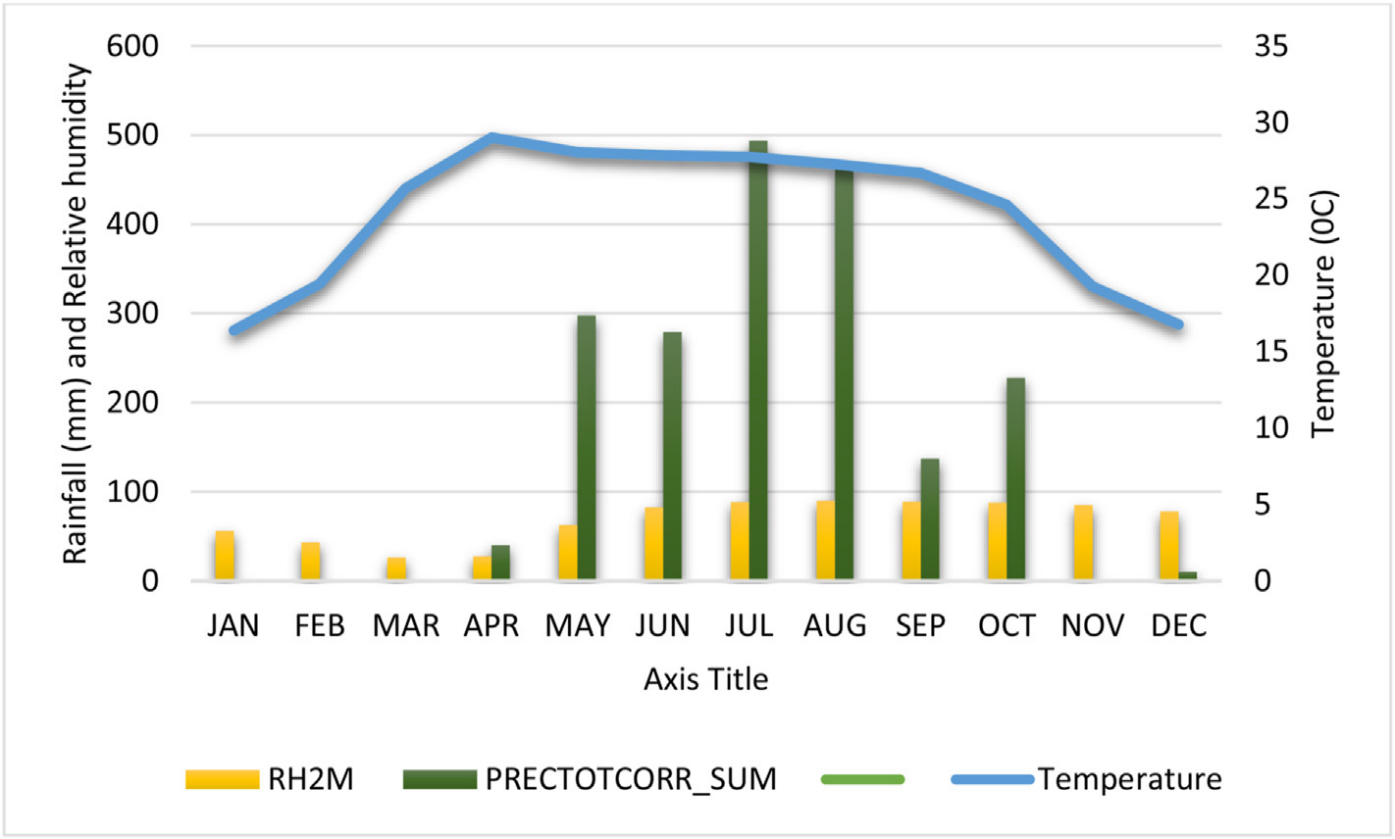
Agro-climatic condition during study period.

The experiment was conducted in the farmers field of Itahari sub-metropolitan municipality (26°39′47″N 87°16′28″E). The study area lies in the terai region of Nepal located at 110 masl that has humid weather with cold winters and very hot summers. The climatic details of the experimental site are presented in Figure 1. The soil type present in study area was clay loam soil. Experiment was conducted in the same field for both the seasons.

### 2.2 Design of experiment and experiment details

The experiment was conducted in Completely Randomized Bock design (RCBD) with 11 genotypes and 3 replications in both the seasons (Summer 2021 and Winter 2021/2022). The genotypes used in the study were collected from local agrovets of Itahari and Biratnagar in their original sachet of 2kg quantity and all of them were commercial maize hybrids developed by various multinational companies. A total of nine multinational company hybrids, RH-10 (developed by NMRP, Chitwan used as a standard check) and Arun 2 (open pollinated variety) as local check was used in the experiment. The details of the genotypes used has been presented in Table 1.

**Table 1:** Details of genotypes used in study.

Treatment code	Genotype name	Company
1	Arun 2	Local Agrovet
2	BS9220	Bio seed research India pvt. ltd.
3	CAH 153 (RH-10)	NMRP, Rampur
4	CP 808	Charoen Popkhand Seeds pvt. ltd.
5	P3553	Du pont pioneer
6	Bisco gold 941	Bisco ​Bio Sciences Private Limited
7	P3355	Du pont pioneer
8	P3396	Du pont pioneer
9	G-25	Genexis Hybrid seeds
10	Rajkumar	Bio seed research India pvt. ltd.
11	TX 369	Bio seed research India pvt. ltd.

The experimental field was laid out plot size of 4 m × 3 m with row to row and plant to plant spacing of 0.75m and 0.2m, respectively. Every genotype was sown in four consecutive rows. The plot to plot distance was 0.75m and block to block distance was 1m. In summer, sowing was done on 25^th^ of April and harvested from 21^st^ of August to 23^rd^ of August. In winter, sowing was done on 15^th^ of November and was harvested from April 18-April 25 when physiological maturity has been attained.

### 2.3 Plant management practices

Field preparation was done by ploughing two times followed by leveling and ensuring optimal moisture for germination. Farm yard manure was applied at the rate of 15 t/ha. Chemical fertilizers were applied at the rate of 180:60:40 kg ha^−1^ of N:P_2_O_5_:K_2_O in the form of Urea, Diammonium Phosphate and Muriate of potash respectively [7]. Planting was done on last week of April, 2021 for summer environment and for winter, on second week of November by line sowing along the row. Phosphorus and Potassium were applied in basal dose and half portion of Nitrogen were applied at the time of sowing respectively. Remaining Nitrogen was applied in two equal splits (25%–25%) after first and second weeding. Two weedings were carried out at 30 DAS and 45 DAS. Thinning out was done during first weeding to ensure single plant per hill and earthing up was done during second weeding.

### 2.4 Data collection

Data collection was done on five randomly selected plants from all plot in both the seasons. Except for reproductive traits, all the traits were recorded after the crop has attained physiological maturity and harvesting. Reproductive traits include day to 50% tasseling (anthesis days), days to 50% silking (silking days) and Anthesis silking interval (ASI). Plant height and ear height was measured at physiological maturity from five randomly selected plants as suggested by Badu-Apraku et al. [12]. After harvesting all the ears from each plot was collected, ear count was taken and later converted in hectare (Equation 1) as suggested by Badu-Apraku et al. [12] which was also followed by others [7, 8, 13, 14, 15, 16] in maize breeding program. Ears from tagged plants were taken and used for recording cob characteristics like Cob length (CL), Cob diameter (CD), Number of grains per row (NOGPR), and Number of rows per cob (NORPC). Grain yield (Equation 2) was calculated after weighing the total harvested ears per plot (Field weight) and grain moisture content in field using the formula as suggested by Badu-Apraku et al. [12]. Thousand kernel weight (Equation 3) was also recorded and later converted into 12.5% moisture content as reference.

(Equation 1) $$NOEPH=\frac{No.of\;ears\;per\;plot\times 10000\;\left(m^{2}\right)}{Plot\;size\;\left(m^{2}\right)}$$

(Equation 2) $$Grain\;yield=\frac{Field\;weight\;\left(kg\right)\times 0.8\times \left(100-Moisture\;content\right)}{Plot\;size\;\left(m^{2}\right)\times 87.5}\times 10$$

(Equation 3) $$1000-kernel\;weight=\frac{Kernel\;weight\times \left(100-moisture\;\%\right)}{100-12.5}$$

### 2.5 Statistical analysis

Data was entered in MS-Excel 2016 and was analyzed using R-studio 4.0.1 and SPSS v.20. Estimation of genetic parameters was done by using R-studio v.4.0.1. Correlation coefficient was estimated using SPSS v.20.

Correlation coefficient was computed by the formula Eq. (1). given by Webster and Moorty and also used by several researchers [17, 18, 19, 20, 21, 22, 23, 24].

(1) $$rp\left(xy\right)=\frac{cov\;p\;\left(xy\right)}{\sigma \frac{1}{2}g\left(x\right)\;*\;\sigma \frac{1}{2}p\left(y\right)}$$

Where.

rp (xy) is phenotypic correlation, cov p (xy) is covariance due to phenotype of xy, and $\sigma \frac{1}{2}g\left(x\right),and\;\sigma \frac{1}{2}p\left(y\right)\;are\;the\;genotypic\;and\;phenotypicstandard\;deviation\;of\;x\;and\;y\text{.}$

As recommended by Singh and Chaudary [25], the phenotypic coefficient of variation as in Eq. (2) and genotypic coefficient of variation (Equation 3) have been determined. Eqs. (4) and (5) measure heritability in broad sense and genetic advance as percentage of mean as suggested by Johnson [26] and Robinson et al. [27], respectively.

(2) $$PCV=\frac{\sqrt{\delta^{2}p}*100}{x}$$

(3) $$GCV=\frac{\sqrt{\delta^{2}g}*100}{x}$$

Where,

$$\delta^{2}p\;is\;variance\;due\;to\;phenotype\;and\;\delta^{2}g\;is\;variance\;due\;to\;genotype\;andx\;is\;the\;population\;mean$$

(4) $$Heritability\;\left(h^{2}bs\right)=\frac{\delta^{2}g}{\delta^{2}p}$$

(5) $$Genetic\;advance\;\left(GA\right)=K\left(\delta^{2}p\right)\left(h^{2}bs\right)$$

Where, K = selection differential that varies depending up on the selection intensity and stands at 2.056 for selecting 5% of the genotypes.

### 2.6 Analysis of variance and AMMI analysis

Data were subjected to analysis of variance across the environments (season) using ADEL-R version 2.0. Additive Main Effect and Multiplicative Interaction (AMMI) model was used for the mean of the yield of the 11 genotype of maize from both the environments using GEA-R software. The AMMI model equation is:

$$Yij=\mu +ai+\beta j+\sum_{n=0}^{N}\lambda n\;\delta in\;ƴjn+\theta ij+\varepsilon ij$$

Where: Yij = the mean yield of elite line i in environment j, μ = the grand mean of the yield, αi = the deviation of the elite lines mean from the grand mean, βj = the deviation of the environment mean from the grand mean, λn = the singular value for the PCA; n, N = the number of PCA axis retained in the model, ƴjn = the PCA score of an elite line for PCA axis n, δjn = the environmental PCA score for PCA axis n, θij = the AMMI residual and = the error term

## 3 Results and discussion

### 3.1 Analysis of variance and mean performance

#### 3.1.1 Plant architectural trait

Analysis of variance revealed that the plant height differed significantly in both the seasons and ear height differed significantly in winter season and was not significantly different in summer season (Table 2). Plants in summer season were comparatively taller than winter season and ear height also showed a similar trend and was also supported by earlier findings of Tripathi et al. [7]. Temperature and solar radiation have direct effect on photosynthesis and inter-node elongation [28, 29, 30, 31]; optimum temperature and sunlight hours are reported in summer season and are comparatively higher than winter season leading to positive effect on plant architectural traits (Figure 1, Table 2). Genotype and environment interaction reveal that there was significant difference in performance of genotypes in two seasons.

**Table 2:** Plant architectural trait in both season.

Genotype	Plant height (cm)		Ear height (cm)	
	Winter	Summer	Winter	Summer
Arun2	205.3d	282.33bcd	107.67cd	138.67b
BS9220	211.33d	290.3bc	99.76de	150.33b
CAH153	208.33d	296ab	97.3de	164.2ab
cp808	255.33a	286.66bc	133a	144.33b
Bisco gold 941	236.43b	288bc	125.33ab	161ab
G-25	236.67b	308.33a	127.33a	202a
P3355	235.23b	257.33f	92.3e	136b
P3396	233.38bc	275cde	114bc	138.33b
P3553	211.28d	265ef	105.41cd	139.33b
Rajkumar	219cd	268.33def	102de	140b
TX369	210.3d	290.67bc	97de	138b
grand mean	223.87	282.61	109.23	143.17
CV	4.22	3.35	6.09	18.31
LSD	16.11	16.17	11.66	46.85
Genotype	∗∗∗	∗∗∗	∗∗∗	Ns
Environment	∗∗∗		∗∗∗	
G x E	∗∗∗		Ns	

CP 808 (255.3cm) was the tallest genotype in winter season followed by Bisco gold 941 (236.6cm), G-25 (236.67cm), and P3355 (235.3cm) (Table 2). In summer season, G-25 (308.3cm) was the tallest genotype followed by CAH 153 (296cm), TX369 (290.67cm) and BS9220 (290.3cm). Highest ear height was reported in CP 808 (133cm) in winter season and G-25 (202cm) in summer season. Plant height and ear height are important traits in maize as they are two main traits for selection in maize architecture because optimal plant height and ear height are critical for improving plant density to maximize fertilizer utilization, water use and incident photosynthetically active radiation [32, 33, 34, 35]. The plant height in the study ranged from 205.33cm- 255.3cm in winter season and 257.3–308.3cm in summer season. The ear height ranged from 92-127cm and 136–164cm in winter and summer season respectively. Several researchers [7, 13, 14, 15, 36] reported plant architectural traits of maize within this range which supports current finding.

#### 3.1.2 Reproductive traits

Analysis of variance revealed that the reproductive traits anthesis days, silking days and anthesis silking interval showed highly significant differences (P ≤ 0.0001) in both the seasons, between the genotypes (Table 3). The anthesis and silking days are significantly higher in winter season than that of summer however the required Growing Degree Days (GDD) was constant in both the seasons [6]. The spring maize are earlier than winter maize for reproductive traits [37] where the average length varies from 100- 111 day in anthesis and 102–113 day in silking in winter season (Table 3). Genotypes P3355 and P3553 were early for days to anthesis and BS9220 took longer time to anthesis in winter season and in summer CP 808 was early for tasseling. Minimum difference in anthesis silking interval was reported in BS9220 and TX369 in winter season and TX-369 in the summer season. In summer season genotypes CAH 153, CP 808 and P 3396 had ASI of one day. The maximum difference was reported in P3553 (6 days) in winter and G-25 in summer season (4.6 days). Anthesis silking interval between 2-4 days are found to have a positive effect on grain yield of maize i.e. ASI is negatively correlated to yield and a close gap in ASI has been reported in maize hybrid by several researchers [23, 37, 38, 39, 40, 41]. The yield and its components showed dependence on the ASI [42]. Wider the gap between tasseling and silking, higher the chances of infertility, unfilled kernels in cob and ultimately reduction in grain yield. The major reason of same genotype having longer silking duration in winter has been attributed to low temperature and sunlight, as silking is highly sensitive to cold stress during crop growth [8].

**Table 3:** Comparison of reproductive traits of both seasons.

Genotype	Anthesis days		Silking days		Anthesis silking interval	
	Winter	Summer	Winter	Summer	Winter	Summer
Arun2	103.33bc	56a	111.33a	57.33ab	5ab	1.3def
BS9220	109.67a	58.33a	108.33ab	60.66a	1.7f	2.33bc
CAH153	101c	55.33a	104.33c	56.33abc	3.3e	1ef
CP 808	95de	47b	99d	48d	4bcd	1ef
Bisco gold 941	97d	49.33b	100d	52.33cd	2.67def	3b
G-25	95.6de	48.66b	100.67d	53.33bc	5ab	4.6a
P3355	87.67f	58.33a	90f	60a	2ef	1.67cde
P3396	94.33e	57a	98.67d	58a	4.33bc	1ef
P3553	88f	58a	94e	59.33a	6a	1.33def
Rajkumar	104.3b	57.66a	107.33bc	59.67a	3cdef	2cd
TX369	104.67b	56.33a	106.33bc	57ab	1.67f	0.67f
Grand mean	98.27	54.72	101.81	56.54	3.54	1.82
CV	1.77	4.6	1.69	4.75	24.6	22.85
LSD	2.96	4.29	3.32	4.57	1.48	0.7
Genotype	∗∗∗	∗∗∗	∗∗∗	∗∗∗	∗∗∗	∗∗∗
Environment	∗∗∗		∗∗∗		∗∗∗	
G x E	∗∗∗		∗∗∗		∗∗∗	

#### 3.1.3 Cob characteristics

Number of rows per cob (NORPC), Number of grains per row (NOGPR) and Cob length (CL) are significantly different in both the seasons. Cob diameter and number of ears per hectare (NOEPH) showed significantly different performance in summer and nonsignificant performance in winter season. The details of the cob characteristics have been presented in Table 4. There were significant differences in performance of genotypes in both seasons for number of rows per cob and number of grains per row but *Genotype × Environment* interaction was non-significant. This might be due to the reason that the trait might be under the genetic control and has low influence on variation in environmental condition [43] and similar finding has also been reported by Dhakal et al. [8] in three-way cross hybrid maize. A significant difference between the genotypes and nonsignificant difference in Genotype × Environment interaction was also reported by Tripathi et al. [7]. The mentioned cob characteristics are the yield attributing traits and are found to be positively correlated with yield i.e. increase in any of the cob characteristics increases the yield [7, 8].

**Table 4:** Comparison of cob characteristics of studied genotypes in both seasons.

Genotype	NORPC		NOGPR		Cob diameter (cm)		Cob length (cm)		NOEPH	
	Winter	Summer	Winter	Summer	Winter	Summer	Winter	Summer	Winter	Summer
Arun2	14cd	14.33de	27de	25.66c	4.13abc	4.17d	17.67de	20defg	57985.33a	48333.33e
BS9220	14cd	14.33de	24.7e	28c	4.37abc	4.42cd	21b	22.67abcd	56894ab	54000cde
CAH153	14.67bcd	13.78e	25.67de	28.4c	4.15abc	4.50cd	16.33de	19.62efg	56829.67ab	55494.56bcd
cp808	14cd	16abc	24.7e	26.89c	3.83c	4.32cd	14f	17.1g	56100ab	49444.44de
Bisco gold 941	13.67d	14.89cde	25.3e	25.56c	4.373abc	4.29d	16ef	18.34fg	56000ab	55000bcd
G-25	13.66d	15.56bcd	28.6cd	32.67b	4.43abc	4.75bc	18.47cd	20.29cdef	55584.67ab	59444.44abc
P3355	17a	16.73ab	38a	41.67a	4.34abc	5.42a	24.33a	25.6a	55111ab	60333.33ab
P3396	15.3bc	17.07a	37.67a	41.867a	4.72a	5.19ab	21b	22.67abcd	52683.33ab	61851.67a
P3553	14.67bcd	16.93a	38a	41.6a	3.96bc	5.26a	24.53a	23.93ab	51000ab	59666.67abc
Rajkumar	14.667bcd	14.67de	31bc	32.67b	3.96bc	4.2d	20.33bc	21.66bcde	50650ab	57926.67abc
TX369	15.67ab	15.33cd	32.67b	33.78b	4.53ab	4.13d	22b	23abc	49223.33b	59386.67abc
Grand mean	14.66	15.42	30.3	32.58	4.25	4.61	19.63	21.35354	54369.21	56443.8
CV	6.64	4.88	5.9	7.5	9.34	5.83	6.85	8.19	8.9	6.5
LSD	1.65	1.28	3.05	4.16	0.69	0.45	2.29	2.97	8261.17	6261.7
Genotype	∗∗	∗∗∗	∗∗∗	∗∗∗	ns	∗∗∗	∗∗∗	∗∗∗	NS	∗∗
Environment	∗∗∗		∗∗∗		∗∗∗		∗∗		NS	
G X E	Ns		NS		∗∗		NS		NS	

#### 3.1.4 Yield and thousand kernels weight

Analysis of variance revealed that the grain yield differed significantly in both the seasons, However the average grain yield was higher in the summer season. Similar finding was also reported by Dhakal et al. [8] where the yield of top performing variety is 8.36 t/ha in winter and 9.76 t/ha in summer season. Researchers suggest that the grain yield of maize is higher in summer season as compared to spring due to long sunshine hours [28, 29], hot and humid climate and low susceptibility to low temperature and highly responsive to nitrogenous fertilizer during summer and spring seasons. The average grain yield of top-performing genotypes is higher in both the seasons in our study as compared to [8]. In winter season, genotype P3355 was high yielding followed by Bisco gold 941, and P3396 and Arun 2 being the lowest producing variety (Table 5). In summer season, P3553 being the highest yielding genotype followed by P3355 and Bisco gold 941, and Arun 2 being the lowest performing genotype. Comparing yield of both the seasons, genotype P3355 and Bisco gold 941 can be suggested for cultivation in both the seasons for terai domain. There was significant difference among the genotypes for 1000-kernel weight during winter season and they were nonsignificant in summer season. Highest 1000-kernel weight was reported in P3355 in winter season and P3553 in summer season.

**Table 5:** Yield comparison and thousand kernel weight in both seasons.

Genotype	Grain yield (t/ha)		Thousand kernel weight	
	Winter	Summer	Winter	Summer
P 3355	9.94a	11.94a	327.33a	297.23a
Arun 2	4.13d	5.93e	248ef	279.6abcd
BS9220	7.76bc	8.66cd	268.71cde	273.67abcd
CAH 153	7.8bc	10.139abc	270.67cd	296.37ab
CP 808	4.21d	7.18de	235f	229.69cd
P 3553	8.59abc	12.21a	284.33bc	332.67a
Bisco gold 941	8.87ab	11.42ab	302.67b	287.38abc
P 3396	8.71ab	10.38bc	284.04bc	268.54abcd
G-25	8.52abc	10.59abc	249.47def	266.44bcd
Rajkumar	7.86bc	9.26bcd	246.34f	268.33abcd
TX-369	6.79c	8.68cd	212.21g	220d
Grand mean	7.56	9.67	266.25	274.54
CV	14.3	13.25	4.71	13.84
LSD	1.85	2.18	21.78	64.74
Genotype	∗∗∗	∗∗∗	∗∗∗	NS
Environment	∗∗∗		NS	
G X E	NS		NS	

### 3.2 Estimation of genetic parameters

#### 3.2.1 Genetic variability

Genetic parameters differed significantly in both the summer and winter seasons. PCV was larger than GCV in both the seasons in terms of all genetic parameters, i. e environment influenced in expression of traits. Similar finding was also reported by several researchers [24, 44, 45]. The PCV and GCV was found highest in terms of ASI with 45.31 and 38.03 respectively in winter season; also, highest PCV and GCV for ASI 66.88 and 62.85 respectively was recorded in summer season (Table 6). Moderate PCV and GCV was found in traits like NOGPR, CL and GY with 18.77, and 17.81, 18.37 and 17.04, 27.19 and 23.08 was observed during winter season respectively. Moderate PCV and GCV was found in traits like NOGPR and GY with 20.64 and 19.23, 23.01 and 18.8 was observed in summer season. Low PCV and GCV was found in terms of PH, EH, AD, SD, NORPC, CD, NOEPH and TKW with 8.03 and 6.82, 13.66 and 12.13, 7.28 and 7.06, 6.5 and 6.21, 8.77 and 5.72, 10.05 and 3.11, 11.02 and 6.48, 12.85 and 11.93 respectively was observed in winter season. Low PCV and GCV was found in terms of PH, AD, SD, NORPC, CL, CD and NOEPH with 5.96 and 4.92, 8.62 and 7.29, 7.89 and 6.28, 8.39 and 6.83,13.60 and 10.86, 11.38 and 9.77, 9.51 and 6.93, respectively in winter season. While in summer season, moderately high PCV was found in terms of EH and TKW with 19.88 and 16.01 respectively; low GCV was found in terms of EH and TKW with 7.74 and 8.04 respectively. The greater variation in PCV and GCV in traits reveal that the expression of trait is heavily influenced by environmental factors [24].

**Table 6:** Estimate of Genetic variability.

Traits under study	Winter		Summer	
	GCV	PCV	GCV	PCV
Plant height	6.82	8.03	4.92	5.96
Ear height	12.13	13.66	7.74	19.88
AD	7.06	7.28	7.29	8.62
ASI	38.03	45.31	62.85	66.88
SD	6.21	6.5	6.28	7.89
NORPC	5.72	8.77	6.83	8.39
NOGPR	17.81	18.77	19.23	20.64
CL	17.04	18.37	10.86	13.60
CD	3.11	10.05	9.77	11.38
NOEPH	6.48	11.02	6.93	9.51
TKW	11.93	12.85	8.04	16.01
GY	23.08	27.19	18.8	23.01

Overall highest GCV was observed in ASI during winter season and summer season among all the genetic parameters. Though, GCV indicates a high level of genetic diversity, but heritability estimates and genetic gain are used to determine the amount of heritable variation in traits [24, 45].

#### 3.2.2 Heritability and genetic advance

In winter season, the highest heritability was reported in anthesis days (0.9407) followed by silking days (0.9131), number of grains per row (0.9004), thousand kernel weight (0.8621), cob length (0.86070, ear height (0.7892), anthesis silking interval (0.7402), plant height (0.7230) and grain yield (0.7205). The least heritable trait was cob diameter (0.0959). The highest heritability during summer season was reported in anthesis silking interval (0.8832) followed by number of grains per row (0.8677), cob diameter (0.7369), anthesis days (0.7148), plant height (0.6827), grain yield (0.6679), number of rows per cob (0.6621), cob length (0.6377), and silking days (0.6339). Thousand kernel weight and ear height were low heritable traits and number of ears per hectare was moderately heritable. This study reported that, in both the seasons, heritability of trait was low (<30%), moderate (30–60%) and highly heritable (>60%) (Table 7) as proposed by Johnson et al. [26] and reported by several authors [7, 24, 44, 45, 46, 47, 48, 49, 50, 51] in trait study of maize.

**Table 7:** Estimates of heritability and genetic advance.

Traits under study	Winter		Summer	
	Heritability	Genetic advance	Heritability	Genetic advance
Plant height	0.7230	11.96	0.6827	8.38
Ear height	0.7892	22.21	0.1517	6.21
AD	0.9407	14.11	0.7148	12.70
ASI	0.7402	65.74	0.8832	81.68
SD	0.9131	12.31	0.6339	10.30
NORPC	0.4267	7.67	0.6621	11.45
NOGPR	0.9004	34.82	0.8677	36.90
CL	0.8607	32.57	0.6377	17.87
CD	0.0959	1.7	0.7369	17.28
NOEPH	0.3454	7.84	0.5315	10.41
TKW	0.8621	22.31	0.2522	8.31
GY	0.7205	40.36	0.6679	31.66

Genetic advance as a percentage of mean ranged from 1.7% in cob diameter to 65.74% in anthesis silking interval in winter season and 6.21% in ear height to 81.68% in anthesis silking interval in summer season. Greater estimates of GAM were reported in anthesis silking interval (65.74) followed by grain yield (40.36), number of grains per row (34.82), cob length (32.57), 1000-kernel weight (22.31), and ear height (22.21) in winter season and in summer season higher estimate of GAM was reported in anthesis silking interval (81.68) followed by number of grains per row (36.9) and grain yield (31.66). Based on heritability and genetic advance of different traits in both season anthesis silking interval, number of grains per row and grain yield are under genetic control governed by additive genes. Mohana Krishna et al. [43] reported that high heritability coupled with high GAM is due to additive effect of genes and, high heritability with low GAM signifies the non-additive genetic effect which supports our finding. In line to our finding, Thapa et al. [24] also reported high heritability and GAM for grain yield.

### 3.3 Correlation analysis

Correlation analysis showed that grain yield is positively and significantly correlated with thousand kernel weight (TKW) (r = 0.597), anthesis silking interval (r = 0.446), plant height (r = 0.381), ear height (r = 0.384), number of grains per row (r = 0.446), number of rows per cob (r = 0.375), cob diameter (r = 0.431), cob length (r = 0.461) and number of ears per hectare (r = 0.499). Reproductive trait like anthesis days (r = -0.487) and silking days (r = -0.466) are negatively and significantly correlated with grain yield i.e. earlier tasseling and silking days are related with high grain yield in maize (Table 8). Similar finding was reported by several authors that grain yield is positively correlated with cob characteristics and negatively related with reproductive traits like silking days and anthesis days [2, 7, 22, 23, 24, 44, 52, 53, 54, 55]. The correlation between the traits has been presented in Table 8. The selection of these traits would suggest an indirect selection of genotypes for grain yield if there is a positive and strong correlation between them [24]. The findings of Thapa et al. [24] suggest that if ASI is longer in crop, it facilitates vegetative growth thus causing lower yield which contradicts our finding as it is positively correlated with grain yield. This finding also contradicts with finding of few researchers [2, 47, 56].

**Table 8:** Simple Correlation analysis of Yield and Yield attributing traits.

	GY (t/ha)	tkw	AD	ASI	SD	PHT	EHT	NORPC	NOGPR	CD	CL	ear per hectare
GY (t/ha)	1	.597∗∗	-.487∗∗	.446∗∗	-.466∗∗	.381∗∗	.384∗∗	.375∗∗	.446∗∗	.431∗∗	.461∗∗	.499∗∗
tkw		1	-0.156	0.117	-0.128	0.163	0.163	0.064	0.099	0.213	0.119	0.035
AD			1	-.947∗∗	.980∗∗	-.897∗∗	-.701∗∗	-.315∗	-0.206	-.302∗	-0.227	-0.185
ASI				1	-.990∗∗	.850∗∗	.651∗∗	.279∗	0.232	.379∗∗	.309∗	0.218
SD					1	-.880∗∗	-.667∗∗	-.308∗	-0.222	-.355∗∗	-.280∗	-0.199
PHT						1	.784∗∗	0.177	0.031	0.220	0.029	0.129
EHT							1	0.052	-0.064	.243∗	-0.086	0.097
NORPC								1	.695∗∗	.470∗∗	.438∗∗	.355∗∗
NOGPR									1	.541∗∗	.735∗∗	.569∗∗
CD										1	.393∗∗	.421∗∗
CL											1	.522∗∗
ear per hectare												1

∗∗ Correlation is significant at the 0.01 level and.

∗ significant at the 0.05 level.

### 3.4 AMMI analysis of grain yield and yield attributing traits in maize

AMMI analysis was done for all the traits under study and it is observed that the trait viz. anthesis silking interval, anthesis days, silking days, plant height, number of ear per hectare, cob diameter, number of rows per cob showed significant variations (p < 0.05) for both the main (genotypes and environment) and interaction effects revealing the presence of considerable variability among the studied genotypes, environment, and their interaction. Genotype was not significantly different for Cob length, number of ears per hectare, ear height, grain yield, number of grains per row and for environment showed a significant variation. Thousand kernel weight showed significant variation between genotypes whereas environment and interaction effects were not significantly different.

In our study, AMMI analysis revealed that maximum part of total variance was attributed to environment followed by genotype and interaction for the traits anthesis days, silking days, anthesis silking interval, ear height, and plant height. Similar finding was reported by Zaid et al. [57]. Maximum part of total variation was attributed to genotype followed by interaction and environment in the traits cob diameter, number of rows per cob, thousand kernel weight. Maximum part of total variation was attributed to genotype followed by environment then interaction has been reported for traits grain yield, number of ears per hectare, cob length, and number of grains per row. Only one Principal component was generated by AMMI analysis and it explained 100% variation of all traits. While comparing maize grain yield under two contrasting seasons on single location Dhakal et al. [8] reported that only one principal component was generated i.e. 100% variation in yield was explained by PC1. Also 100% variation in PC1 in AMMI analysis has also been reported by [58] in comparing yield of wheat in terminal heat stress. The AMMI graph of PC1 and traits under study has been presented in (Supplementary figure).

## 4 Conclusion

Hybrid maize is well known for its remarkable contribution to food security as it out yielded OPV's by more than double their production. However, the hybrids that performed superior in one environment do not perform similarly in other locations and environments. In this study, locally available for sale commercial hybrids were used for evaluation and they reported a significant variation in both the seasons for yield and yield attributing traits. Correlation coefficient revealed that cob characteristics have a significant and positive association with grain yield. The field experiment over two seasons revealed that genotype P3355 and Bisco gold 941 showed promising yield when cultivated in winter and summer seasons under optimum conditions. Farmers direct evaluation for grain yield and yield attributing traits of two recommended hybrids (P3355 and Bisco Gold 941) under various planting geometry and nutrient content is suggested for optimal production and increased profitability.

## Ethics approval and consent to participate

Not applicable.

## Consent for publication

Not applicable.

## Declarations

### Author contribution statement

Sandesh Thapa; Sandhya Adhikari: Conceived and designed the experiments; Performed the experiments; Analyzed and interpreted the data; Wrote the paper.

Sara Rawal: Conceived and designed the experiments; Analyzed and interpreted the data; Contributed reagents, materials, analysis tools or data; Wrote the paper.

### Funding statement

This research did not receive any specific grant from funding agencies in the public, commercial, or not-for-profit sectors.

### Data availability statement

Data will be made available on request.

### Declaration of interests statement

The authors declare no conflict of interest.

### Additional information

No additional information is available for this paper.
